# Supplementary material for: Multi Locus View: an extensible web-based tool for the analysis of genomic data
Source: Commun Biol. 2021 May 25;4:623. doi: 10.1038/s42003-021-02097-y (PMC8149710; doi:10.1038/s42003-021-02097-y)
Supplement: Supplementary file 2 — Supplementary Information [file 42003_2021_2097_MOESM2_ESM.pdf]

Multi Locus View is an extensible web-based-tool for the analysis of genomic data

Martin J Sergeant[1], Jim R Hughes[1,2], Lance Hentges[1], Gerton Lunter[1,3], Damien J Downes[2] and Stephen Taylor[1*]

[1]MRC WIMM Centre for Computational Biology, MRC Weatherall Institute of Molecular Medicine, University of Oxford, Oxford, UK and [2]MRC Molecular Haematology Unit, MRC Weatherall Institute of Molecular Medicine, University of Oxford, Oxford, UK [3]University Medical Centre Groningen, Department of Epidemiology, University of Groningen, The Netherlands

*To whom correspondence should be addressed

Supplementary Information

Supplementary Methods

1. Analysis of data in ENCODE project ENCSR391NPE

Initially the file containing the consensus MACS2 peak calls which include the average $-\log_{10}$ q and p scores (replicated peaks file ENCFF058TAP) was uploaded to MLV. Next, bigWigs of the corresponding alignments, ENCFF025ZEN (orange track) and ENCFF421QFK (brown track) and corresponding inputs, ENCFF483ELD and ENCFF769UET (grey tracks) were added to the browser (see *Adding Tracks*). Images from each location were then generated and a graph showing the $-\log_{10}$ q values contained in ENCFF058TAP were generated (see Adding Images). The data was ordered by this value and the view switched to image mode.

2.Functional annotation of regulatory elements

Bam files from alignments of H3K4me1, H3K4me3, H3K27ac and CTCF ChIP-seq experiments [1], as well as ATAC were converted to BigWig files using deepTools[2] *bamCoverage -binSize 50 --normalizeUsingRPKM*. Open chromatin regions were identified by calling peaks from the ATAC bigWig using MACS2 [3] *callpeak -f BAM -g hs* . Peak regions were extending 500 bp either side of the peak summit and any peaks with a maximum height of below 10 were removed.

This file containing 38,537 peaks was uploaded to MLV and then the Peak Stats Function was run on the five bigWig tracks, H3K4me1, H3K4me3, H3K27ac, CTCF and ATAC-seq previously generated. This feature imports the BigWig files into the genome browser and calculates the maximum height, area, and density (area/region width) for each location. Images from each location were then generated (see Adding Images). The Find TSS Distances feature was used to calculate the distance to the nearest annotated transcription start site (TSS) for each region, annotating if the region overlapped a TSS with a TRUE or FALSE value (Fig. 2bv and cv). Using the method of Kowalczyk *et al* [1] the ratio of H3K4me1 to H3K4me3 was added (Fig. 2 biii and ciii - see Adding Columns). Using both the max height and area for peaks in all five chromatin datasets, dimension reduction with both t-SNE and UMAP was performed using the Cluster on Columns feature. This produced two scatter plots for UMAP and t-SNE (Fig. 2 bi,ii and ci,ii), which were colored by CTCF density and TSS overlap respectively (see Scatter Plots). Finally a histogram of H3K27ac peak density (fig 3Biv and Civ).

3.Analysis of cohesin/CTCF interactions

The four narrowPeak files (GEO GSE126634) from CTCF and SCC1 in the WT and CTCF mutant cell lines were concatenated and merged keeping the maximum -log10 qscore and signal value using *bedtools merge --c 5,6 -o max,max*. The corresponding BigWig files were re-created from the original fastq files (SRA SRP18610) according to the methods outlined in the paper [4] except the *minMappingQuality* parameter was removed from the *bamCoverage* command.

The bed file containing all the 104799 merged peaks was uploaded to MLV and the Peak Stats Function was run on the four bigWig Files. Next two columns were added containing the log2 fold difference of CTCF and SCC1 between the CTCF mutant and WT, with the Add Column feature, which also added histograms from these columns (Fig 3. ai,ii and bi,ii). Then regions which overlapped with black listed regions, taken from ENCODE (ENCSR636HFF) were calculated using the Annotation Overlap feature (Fig. 3 b iii) . Finally images of the four BigWigs from each location were generated. Those locations with a max peak height for CTCF in WT cells greater than 100 were tagged as ‘high CTCF’ and the rest as ‘low CTCF’ (Fig. 3 biv). H3K4me3, H3K27me3 and H2AK119ub1 ChIP-seq data from Hap1 cells [5] was added to the project by using the ‘peak stats’ feature on the BigWig files associated with GSM2978163, GSM2978165, GSM2978167. The data was visualized by adding a column average bar chart of the H3K4me3, H3K27me3 and H2AK119ub1 peak area (supplementary Fig. 2)

Supplementary Figures

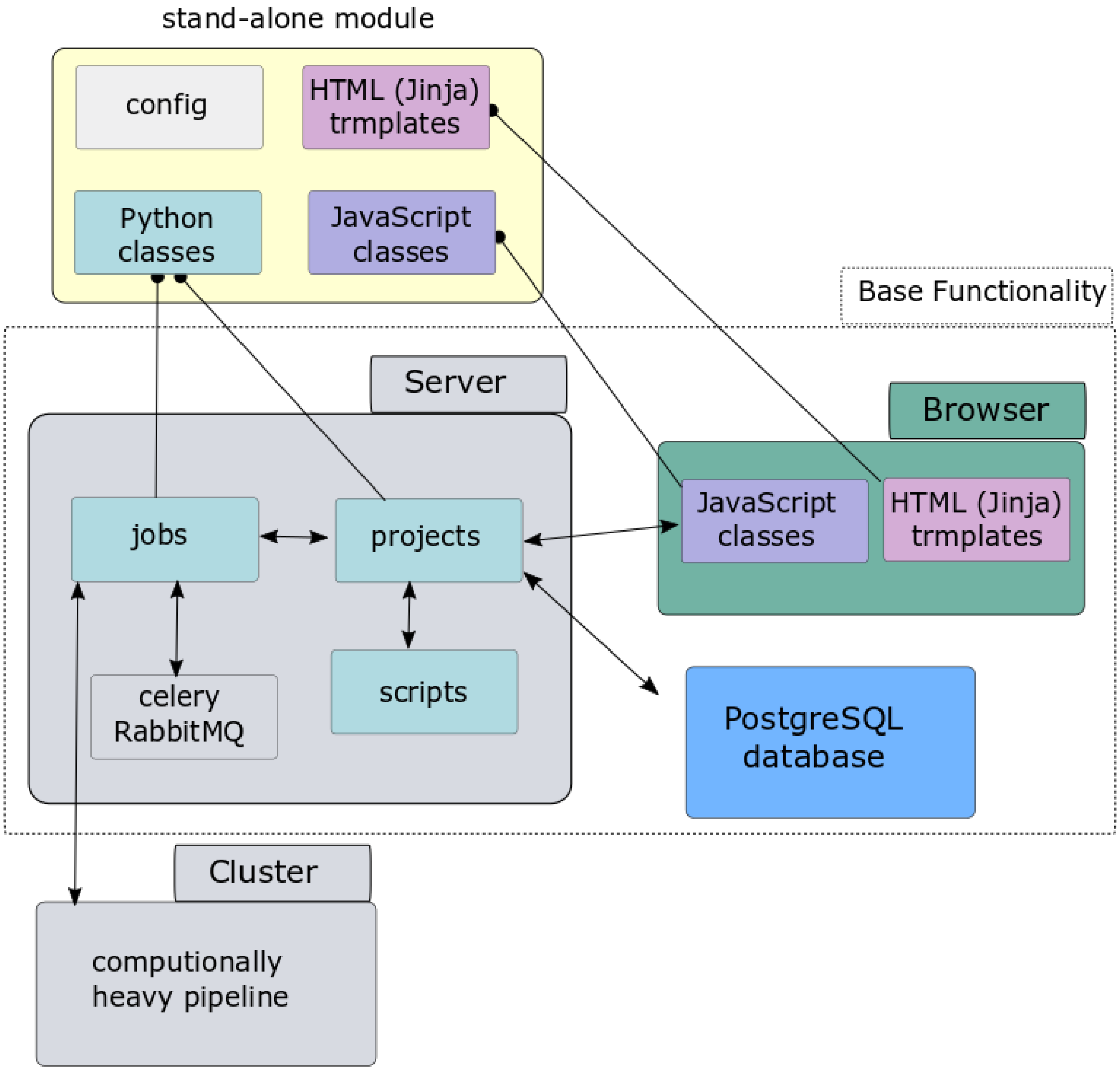

Supplementary Figure 1. Summary of the architecture of MLV

The backend is written in Python and consists of two main class types, jobs and projects. Projects (analysis types) contain methods for interacting with the application (API) and can be accessed directly via python scripts or through http via a single flask view (method), which checks permissions etc. before calling the appropriate project method. Jobs are responsible for running pipelines and tasks either locally using celery via the rabbitmq message queue or remotely on other servers/clusters and are controlled by the projects. Jobs and Projects store their data in a PostgreSQL database. The frontend consists of HTML (Jinja) templates and Javascript classes, which communicate with the projects via ajax calls. Base Python/JavaScript classes as well as Jinja templates contain all the generic functionality. Modules involve extending these classes and templates to tailor the functionality to a particular analysis and are completely stand alone, in that they can be developed independently and added or removed without affecting other modules.

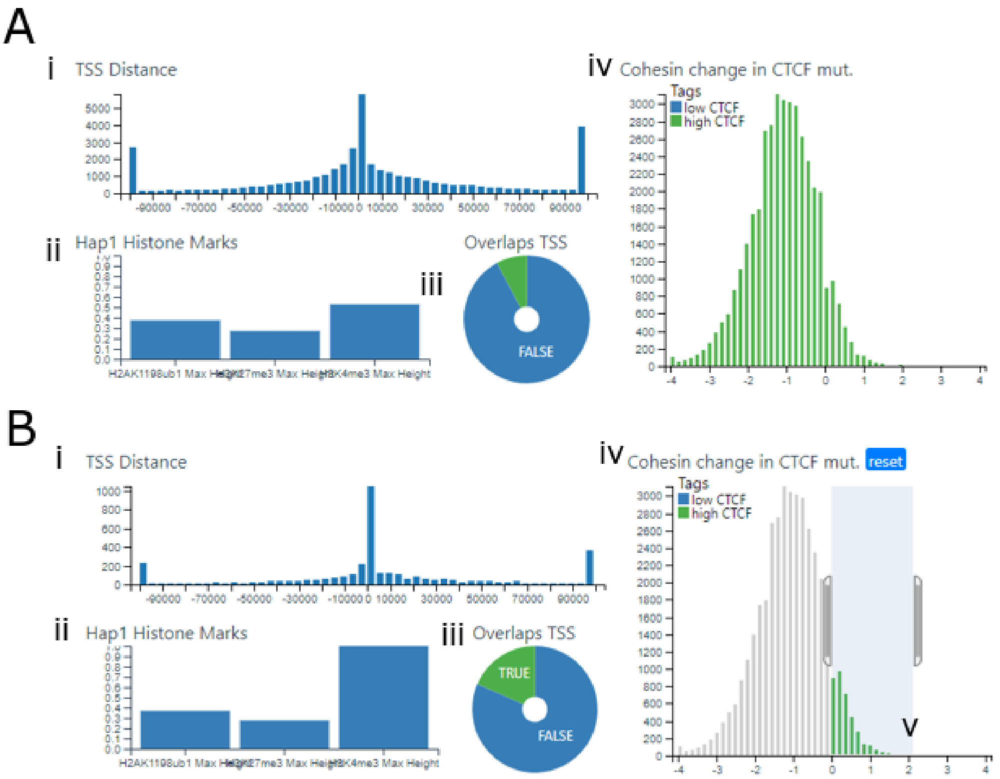

Supplementary Figure 2. Graphs showing differences in TSS overlap/distance and H3K27me3 binding in regions where SCC1 (cohesin) binding is increased in the CTCF mutant [4].

(A) All Regions which strongly bind CTCF (excluding black listed regions) **(B)** Further selection of regions where cohesin increases in the CTCF muttant **(i)** Histogram showing the distance from TSSs of the selected regions **(ii)** bar chart showing the relative average of H2AK119ub1, H3K27me3 and H3K4me3 peak area at the selected locations, **(iii)** Pie chart showing number of selected regions which overlap TSS sites **(Iv)** histogram showing log fold change of cohesin binding (SCC1 ChIP-seq peak height) in the CTCF mutant compared to the WT.

Supplementary References

1. Kowalczyk, M. S. *et al.* Intragenic enhancers act as alternative promoters. *Mol. Cell* **45**, 447–458 (2012).
2. Ramírez, F. *et al.* deepTools2: a next generation web server for deep-sequencing data analysis. *Nucleic Acids Res.* **44**, W160–5 (2016).
3. Gaspar, J. M. Improved peak-calling with MACS2. *bioRxiv* (2017) doi:10.1101/496521.
4. Li, Y. *et al.* The structural basis for cohesin-CTCF-anchored loops. *Nature* **578**, 472–476 (2020).
5. Campagne, A. *et al.* BAP1 complex promotes transcription by opposing PRC1-mediated H2A ubiquitylation. *Nat. Commun.* **10**, 348 (2019).
